# Facile Synthesis of Manganese Cobalt Oxide/Nickel Cobalt Oxide Composites for High-Performance Supercapacitors

**DOI:** 10.3389/fchem.2018.00661

**Published:** 2019-01-17

**Authors:** Wang Chen Huo, Xiao Li Liu, Yun Song Yuan, Nan Li, Tian Lan, Xiao Ying Liu, Yu Xin Zhang

**Affiliations:** ^1^State Key Laboratory of Mechanical Transmissions, College of Materials Science and Engineering, Chongqing University, Chongqing, China; ^2^College of Urban Construction and Environmental Engineering, Chongqing University, Chongqing, China; ^3^Aerospace Institute of Advanced Materials & Processing Technology, Beijing, China; ^4^Engineering Research Center for Waste Oil Recovery Technology and Equipment, Ministry of Education, College of Environment and Resources, Chongqing Technology and Business University, Chongqing, China

**Keywords:** transition metal oxides, spinel structure, composites, supercapacitor, MnCo_2_O_4.5_@NiCo_2_O_4_ nanowire

## Abstract

Transition metal oxides (TMOs) with spinel structures have a promising potential as the electrode materials for supercapacitors application owning to its outstanding theoretical capacity, good redox activity, and eco-friendly feature. In this work, MnCo_2_O_4.5_@NiCo_2_O_4_ nanowire composites for supercapacitors has been successfully fabricated by using a mild hydrothermal approach without any surfactant. The morphology and physicochemical properties of the prepared products can be well-controlled by adjusting experimental parameters of preparation. The double spinel composite exhibits a high specific capacitance of 325 F g^−1^ (146 C g^−1^) and 70.5% capacitance retention after 3,000 cycling tests at 1 A g^−1^.

## Introduction

The rising concerns about environmental crisis and increasing demand of renewable energy sources have attracted extensive attention for developing a secure, high-performance and sustainable storage technology (Lukatskaya et al., 2016; Zhang et al., 2018a). Recently, various energy storage technologies have emerged, such as lithium ion batteries (Zheng et al., 2018), Li-ion sulfur batteries (Sun et al., 2018) and supercapacitors (Qu et al., 2017) etc. Supercapacitors, also named as electrochemical capacitors (ECs), will be one of the most desirable power devices of next generation, owing to their high power density (Simon and Gogotsi, 2008), rapid charging-discharging rate (Miller and Simon, 2008), and excellent cycle stability (Dai et al., 2018). Various materials have been investigated as electrodes for ECs, such as carbon materials (Zhao et al., 2010; Niu et al., 2013; Zhang et al., 2018b), transition metal oxides (TMOs) (Liu et al., 2012; Nam et al., 2012) and conductive polymers (Lim et al., 2015). Generally, TMOs are superior in specific capacitance and stability. Their specific capacitance is 10~100 times of carbon materials, and they have better stability than conductive polymers. Among the TMOs, the spinel structures, especially spinel ternary TMOs, including the MnCo_2_O_4.5_ (Li et al., 2014), NiCo_2_O_4_ (Wang C. et al., 2016), and ZnCo_2_O_4_ (Wu et al., 2015) etc., have been extensively investigated and exhibited an excellent electrochemical performance as electrode materials for ECs, due to its extremely high theoretical specific capacitance, good redox activity, and eco-friendliness (Qiu et al., 2018; Parveen et al., 2019), thus implying that these could be the most potential electrode materials for next-generation ECs.

Over the past few years, numerous researchers have been devoted to develop new strategies to enhance the electrochemical performance of NiCo_2_O_4_ nanomaterials for ECs electrodes (Sun et al., 2016). For example, Wang C. et al. (2016) reported that the NiCo_2_O_4_ nanoneedle was directly anchored on the Ni foam and carbon fabrics, respectively, via a facile hydrothermal method following with a calcination process in air. This strategy would enhance the combination of electrode material and substrate for improving the electrical conductivity and facilitating electrochemical performance. Sun et al. (2016) found that the porous NiCo_2_O_4_ nanograss supported on Ni foam shown a surprisingly high specific capacitance of 807.7 F g^−1^ at 1 mA cm^−2^ (0.38 A g^−1^) after suffering the hydrogenation process for 3 h, which is ascribed to the formation of oxygen vacancies in disordered surface layers during the hydrogenation process and enhance the electrical conductivity. And there are many other strategies to improve the agglomeration of nanomaterials and facilitating contacting of electrolyte and electrode to promote the performance, such as NiCo_2_O_4_@3DNF framework (Parveen et al., 2019) and Co_9_S_8_@NiCo_2_O_4_ nanobrushes (Liu et al., 2019) etc. However, the price of Ni is eight times than the Mn (Information comes from the SMM Information & Technology Co, Ltd.), the high cost severely restricted its commercial applications. Hence, utilizing Mn to substitute Ni or constructing the Mn-Co-O@Ni-Co-O double spinel composites, should be a feasible and effective method for reducing the cost and promoting its application. Unfortunately, the Mn substituted spinel structure always exhibited the poor specific capacitance (Li et al., 2014, 2015; Hao et al., 2015; Wang K. et al., 2016). Thus, fabricating the Mn-Co-O@Ni-Co-O double spinel composites would be an alternative preferable strategy, which is not only decreasing the commercial applications cost, but also improving the electrochemical performance of double spinel composites for ECs via utilizing the synergistic effect of the core and shell.

Herein, the core-shell MnCo_2_O_4.5_@NiCo_2_O_4_ double spinel structures were successfully synthesized by a facile hydrothermal route without any surfactant, where the core, hierarchical MnCo_2_O_4.5_ nanowires with a diameter of 300~500 nm was provided by Li et al. (2015). The prepared MnCo_2_O_4.5_@NiCo_2_O_4_ hybrids exhibit a promising electrochemical performance via comparing with literature results of selected samples which have similar components and structures (Table 1) (Wu et al., 2011; Kuang et al., 2014; Li et al., 2014, 2015; Hao et al., 2015; Sun et al., 2016; Wang C. et al., 2016; Wang K. et al., 2016). This strategy not only diminishes the dosage of Ni, but also elevates the electrochemical performance of the double spinel composites for ECs, which are favor of the commercial applications for next generation energy storage devices.

**Table 1:** Comparison of specific capacitances of selected literature results obtained from materials with similar components and this work.

**Samples**	**Electrolyte**	**Test condition**	**Cs (F g^**−1**^)**	**References**
MnCo_2_O_4.5_	1 M Na_2_SO_4_	0.5 A g^−1^	80.2 F g^−1^	Li et al., 2015
MnCo_2_O_4.5_	1 M KOH	1 A g^−1^	118.8 F g^−1^	Li et al., 2014
MnCo_2_O_4.5_	1 M Na_2_SO_4_	2 A g^−1^	114 F g^−1^	Wang C. et al., 2016
Carbon aerogel@MnCo_2_O_4.5_	1 M Na_2_SO_4_	0.2 A g^−1^	269.9 F g^−1^	Hao et al., 2015
NiCo_2_O_4_@Ni foam	6 M KOH	1 mA cm^−2^	807.7 F g^−1^	Sun et al., 2016
NiCo_2_O_4_@Ni foam	2 M KOH	1 A g^−1^	655 F g^−1^	Wang K. et al., 2016
NiCo_2_O_4_	2 M KOH	1 A g^−1^	372 F g^−1^	Kuang et al., 2014
NiCo_2_O_4_	1 M KOH	1 mA cm^−2^	217 F g^−1^	Wu et al., 2011
MnCo_2_O_4.5_@NiCo_2_O_4_	3 M KOH	1 A g^−1^	325 F g^−1^	This work

*All values are derived from characterizations in three-electrode systems*.

## Experimental Section

### Materials Synthesis

**Figure 1:**
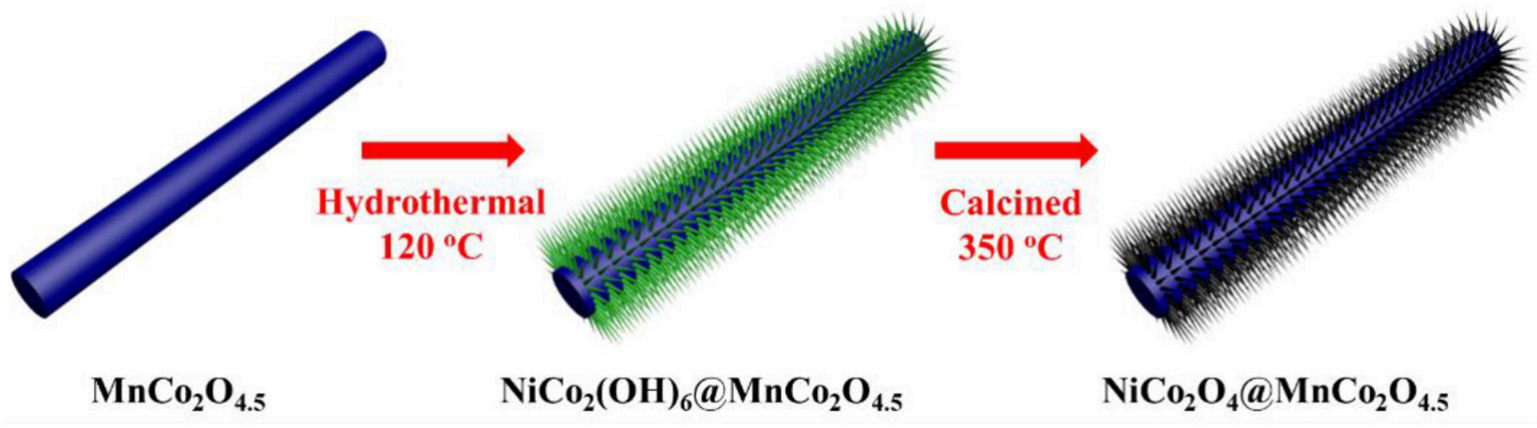
Schematic illustration of the procedure to synthesize NiCo_2_O_4_@ MnCo_2_O_4.5_ composites.

MnCo_2_O_4.5_ nanowires provided by Li's group were adopted as the reaction substrates in the experimental. The synthesis procedure of MnCo_2_O_4.5_ has been reported in detail in Li's work (Li et al., 2015). Figure 1 shows the schematic illustration of the MnCo_2_O_4.5_@NiCo_2_O_4_ nanowires, and the composites were fabricated as follows: 0.001 mol Ni(NO_3_)_2_·6H_2_O, 0.002 mol Co(NO_3_)_2_·6H_2_O, 0.002 mol NH_4_F and 0.005 mol urea were dissolved in 35 mL deionized water at room temperature, then 20 mg MnCo_2_O_4.5_ were added into the mixture, supplemented by ultrasonic treatment until the MnCo_2_O_4.5_ powder were dispersed uniformly. Then the suspension was put in the Teflon-lined stainless autoclave, sealed and heated at 120°C for 6 and 12 h, respectively. After cooling down to room temperature, collected precipitates were washed several times using ethanol and deionized water to remove the attached reaction products and/or residual reactants, then put into the vacuum oven at 60°C to dry. Finally, the metal hydroxides were calcined at 350°C for 2 h to convert into metal oxides most likely as described by the following equations (Xu and Wang, 2011):

(1) $$\begin{array}{r} Ni^{2+}\;+\;2Co^{2+}\;+\;6OH^{-}\;\to NiCo_{2}{\left(OH\right)}_{6} \end{array}$$

(2) $$\begin{array}{r} NiCo_{2}{\left(OH\right)}_{6}\;+\frac{1}{2}O_{2}\;\to \;NiCo_{2}O_{4}\;+3H_{2}O \end{array}$$

### Characterization

X-ray photoelectron spectroscopy (Kratos XSAM800, XPS), the powder X-ray diffraction (D/max 2500, Cu Kα, XRD) and thermosgravimetric analyzer-differential scanning calorimeter (NETZSCH STA 449C, TGA-DSC) were used to characterize the crystallinity and components of prepared materials. Morphological structure was analyzed by scanning electron microscopy (Zeiss Auriga FIB/SEM). The detailed structures of the materials were collected by transmission electron microscopy (FEI TECNAI G2 F20, TEM).

### Electrochemical Measurement

In this work, the three-electrode configuration with the potentiostat/galvanostat model (CHI660E electrochemical workstation) was employed to detect the electrochemical performance of the prepared electrode materials, where nickel foam (1 × 1 cm) supported MnCo_2_O_4.5_@NiCo_2_O_4_ composites worked as the working electrode, saturated calomel electrode (SEC) served as the reference electrode and platinum plate acted as the counter electrode in 3 M KOH electrolyte. The materials for the preparation of working electrode are active materials, acetylene black and polyvinylidene difluoride (PVDF) (mass ratio, 7:2:1). After calculation, the net mass of MnCo_2_O_4.5_@NiCo_2_O_4_ loaded on each working electrode is 1.5 mg.

The cyclic voltammetry (CV) and charge-discharge (GCD) measurements were tested on a CHI660E electrochemical workstation. The CV curves were carried out at disparate scan rates between 0 and 0.5 V (vs. SCE), while the charge-discharge curves (0~0.45 V vs. SCE) were monitored at different current densities. The electrochemical impedance spectroscopy (EIS) plots were obtained over the frequency from 10^−2^ to 10^5^ Hz with the 5 mV amplitude. The normalized specific capacitance (***C***, F g^−1^) and (***Q***, C g^−1^) was calculated from charge-discharge curves and following the equation (Dai et al., 2017):

(3) $$\begin{array}{r} \text{C}=\text{I}\Delta \text{t/}\Delta \text{Vm} \end{array}$$

(4) $$\begin{array}{r} \text{Q}=\text{I}\Delta \text{t/m} \end{array}$$

where *I* is the operated current (A), Δ*t* the discharge time (s), *m* the mass of active electrode materials (g), and Δ*V* the potential window of discharge (V).

## Results and Discussion

**Figure 2:**
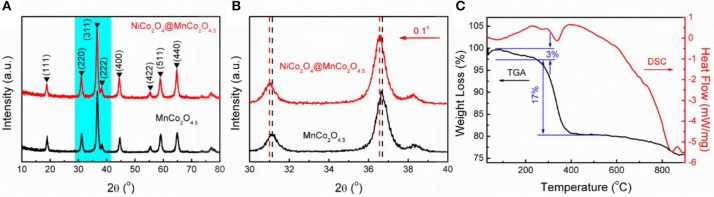
**(A)** XRD patterns of MnCo_2_O_4.5_ nanowires and MnCo_2_O_4.5_@NiCo_2_O_4_ composites. **(B)** The enlarged XRD patterns at the 2θ of 30–40. **(C)** TGA-DSC curves of Ni-Co hydroxide@MnCo_2_O_4.5_ composites.

**Figure 3:**
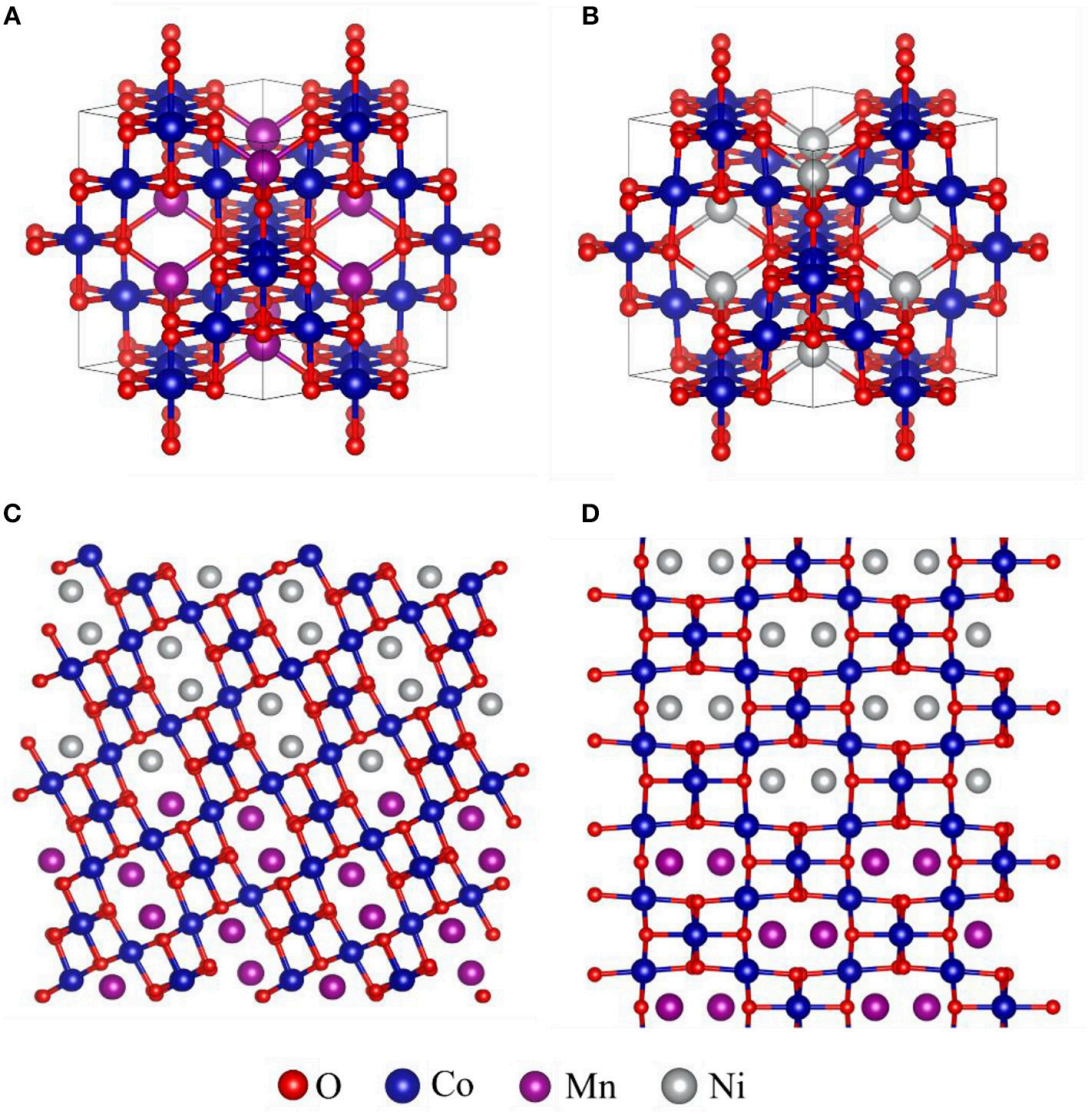
The schematic diagram of MnCo_2_O_4.5_ **(A)** and NiCo_2_O_4_ **(B)** crystal structure. Structure description for possible epitaxial growth patterns of MnCo_2_O_4.5_@NiCo_2_O_4_ **(C)** NiCo_2_O_4_ growing along (311) plane of MnCo_2_O_4.5_; **(D)** NiCo_2_O_4_ growing along (440) plane of MnCo_2_O_4.5_.

The X-ray diffraction peaks of the prepared products were well-indexed to the cubic phase of MnCo_2_O_4.5_(JCPDS 32-0297) (Figure 2A) (Hao et al., 2015). No additional peak for other phase of MnCo_2_O_4.5_ was observed and the sharp and narrow diffraction peaks forecast that the MnCo_2_O_4.5_ microstructures have high crystallinity. The diffraction peaks of the MnCo_2_O_4.5_@NiCo_2_O_4_ have minor left shift (0.1°) compared with MnCo_2_O_4.5_ (Figure 2B), which is ascribed the crystallinity of NiCo_2_O_4_ (JCPDS 20-0781) is very similar to MnCo_2_O_4.5_ (Sun et al., 2016; Wang C. et al., 2016), but the lattice constant is tiny bigger than MnCo_2_O_4.5_. NiCo_2_O_4_ and MnCo_2_O_4.5_ are both spinel structured crystals and have a high degree of lattice matching (~0.3% mis-match). The lattice parameters of MnCo_2_O_4.5_ are 8.08^*^8.08^*^8.08 < 90.0^*^90.0^*^90.0>, and NiCo_2_O_4_ 8.11^*^8.11^*^8.11 < 90.0^*^90.0^*^90.0> and the Figures 3A,B show the crystal structure of MnCo_2_O_4.5_ and NiCo_2_O_4_, respectively. Moreover, in the XRD results the peak intensities of the (311) and (440) planes are higher than others, therefore NiCo_2_O_4_ is most likely to grow along the (311) and (440) planes and completely coherent with MnCo_2_O_4.5_ at the (311) and (440) planes. Figures 3C,D shows the possible epitaxial growth patterns of MnCo_2_O_4.5_@NiCo_2_O_4_ as mentioned above.

Thermogravimetric (TGA) and differential scanning calorimetry (DSC) were performed at a 10°C min^−1^ heating rate to investigate the thermal properties (Figure 2C). The TGA-DSC curve of the Ni-Co hydroxide on MnCo_2_O_4.5_ is shown in Figure 2C. The evaporation of the physically adsorbed water resulted in the weight loss (about 3%) till 280°C. And the followed 17% weight loss between 280 and 400°C is most likely to be caused by the crystalliferous water loss and the decomposition of Ni-Co hydroxide. There is no obvious weight loss after 400°C. Based on these findings, 350°C is chosen and believed to be sufficient for calcination treatment of hydroxides.

**Figure 4:**
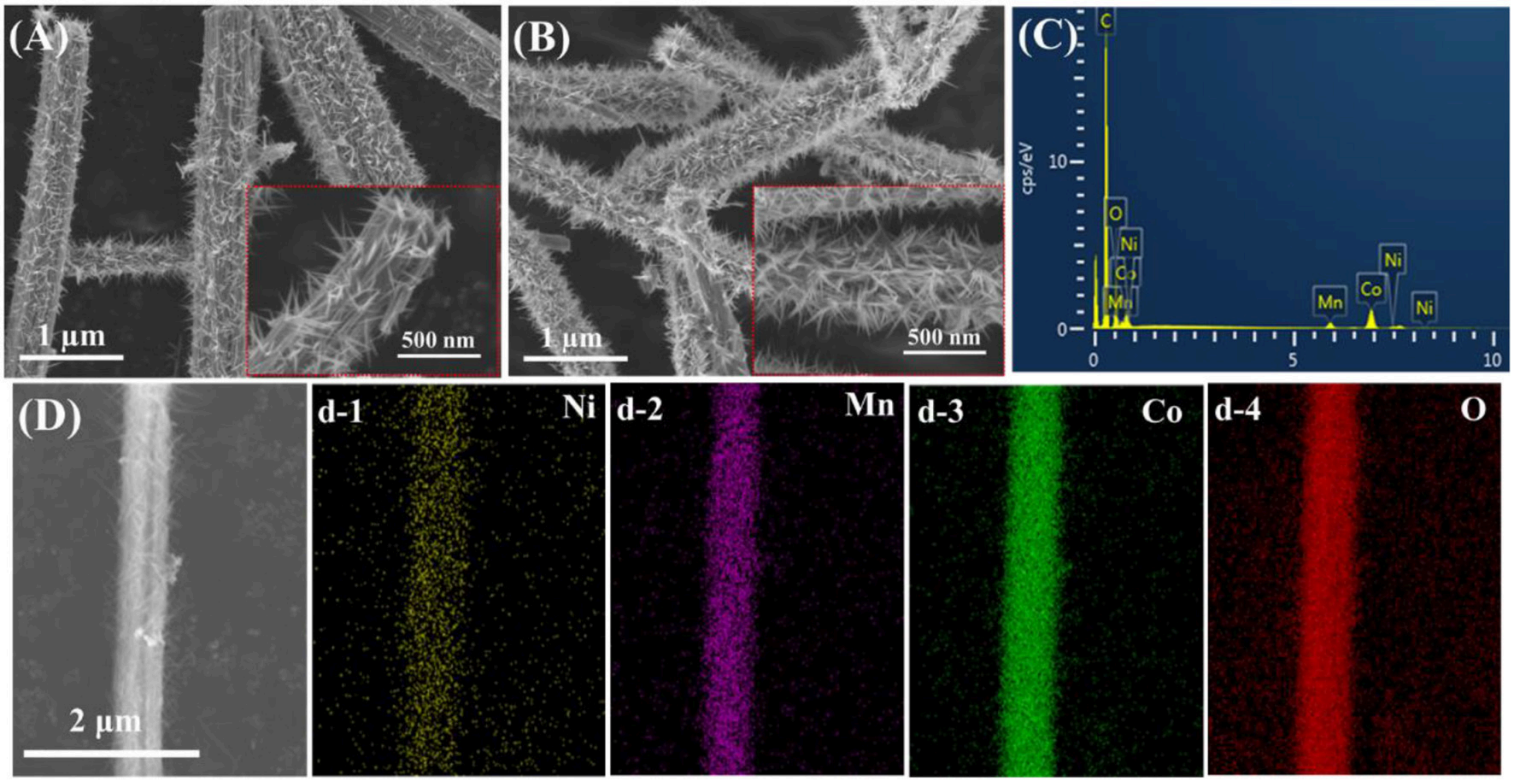
SEM images of **(A)** MnCo_2_O_4.5_@NiCo_2_O_4_-6h and **(B)** MnCo_2_O_4.5_@NiCo_2_O_4_-12h; **(C,D)** EDS mapping results from MnCo_2_O_4.5_@NiCo_2_O_4_.

**Figure 5:**
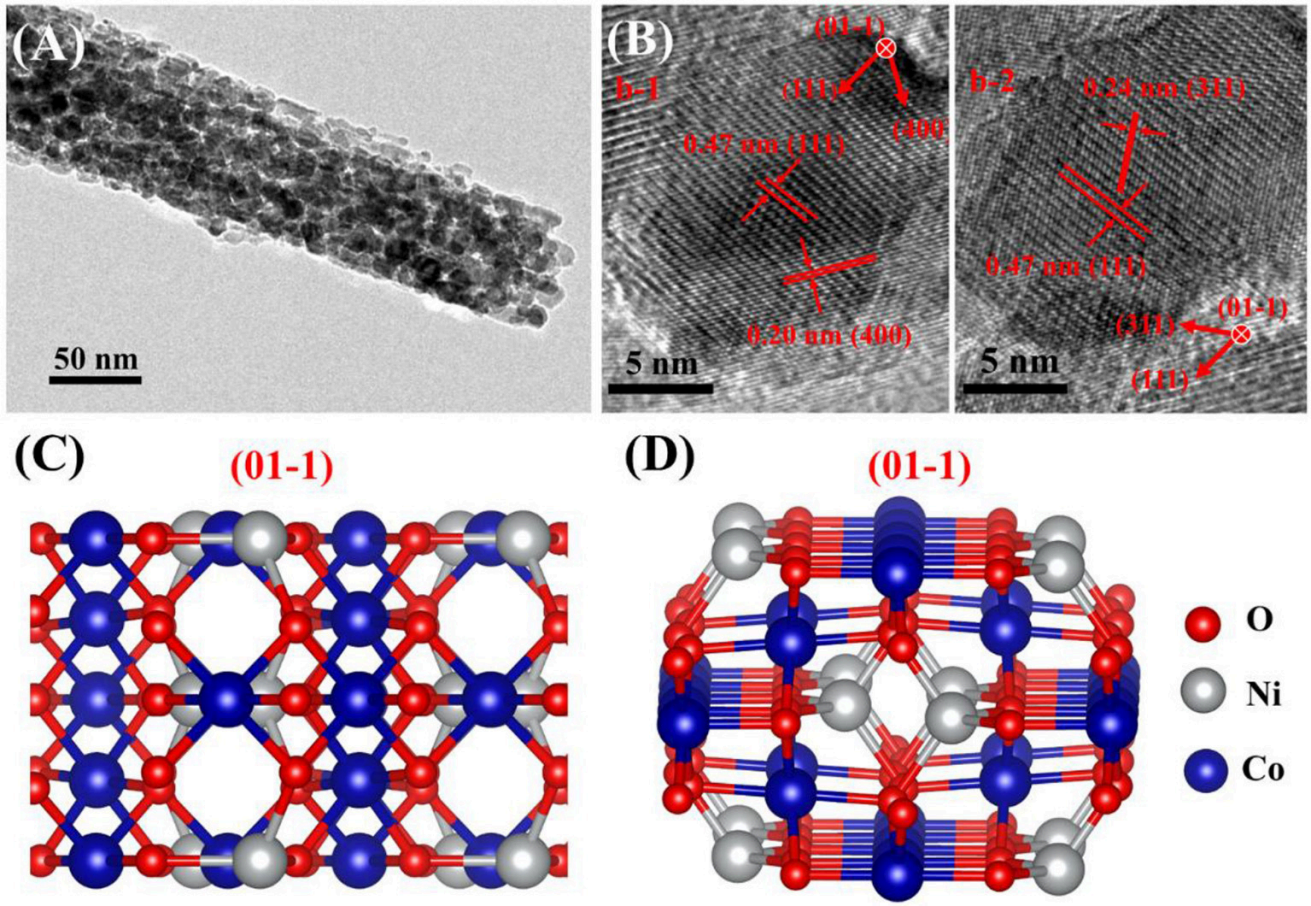
**(A)** TEM and **(B)** HRTEM images of the shell NiCo_2_O_4_ nanowires (**b-1** and **b-2** are the different site of the NiCo_2_O_4_ nanowires); **(C,D)** the schematic diagram of NiCo_2_O_4_ with (01-1) facets exposed.

SEM was adopted to investigate the morphologies of as-prepared composites. As shown in Figure S1, the MnCo_2_O_4.5_ nanowires exhibit fibrous morphologies with smooth surface. The length MnCo_2_O_4.5_ nanowires are several micrometers and the average diameter is about 300~500 nm. Figures 4A,B shows that NiCo_2_O_4_ nanowires with smaller diameter are successfully and uniformly grown on the surface of MnCo_2_O_4.5_ nanowires. When the reaction time increases from 6 to 12 h, dense NiCo_2_O_4_ nanowires are observed (Figure 4B) with more nanowires locating within the unit area of MnCo_2_O_4.5_ surface. The two insets in Figures 4A,B are the images of corresponding samples at higher magnifications. The EDS mapping demonstrates the uniform distribution of Mn, Ni, Co, and O (Figures 4C,D), suggesting successful fabrication of well-distributed MnCo_2_O_4.5_@NiCo_2_O_4_ nanowires and forming the core-shell structure. Furthermore, the detailed structural information of the coated NiCo_2_O_4.5_ shell nanowires was collected by TEM at different magnifications (Figures 5A,B). The primary NiCo_2_O_4_ nanowires have a diameter of 50~80 nm. High-resolution TEM (HRTEM, Figure 5B) image of the NiCo_2_O_4_ nanowires reveal well-resolved lattice fringe having an inter-planar spacing of 0.20 and 0.47 nm (**b-1**), which are well-consistent with the distance of the (400) and (111) plane of NiCo_2_O_4_, respectively. And the (**b-2**) shows inter-planar spacing of 0.24 and 0.47 nm, which represent the (311) and (111) facet of NiCo_2_O_4_. Therefore, these results about the crystal structure and facets illustrate that the (01-1) facets of NiCo_2_O_4_ are exposed (Figures 5C,D).

**Figure 6:**
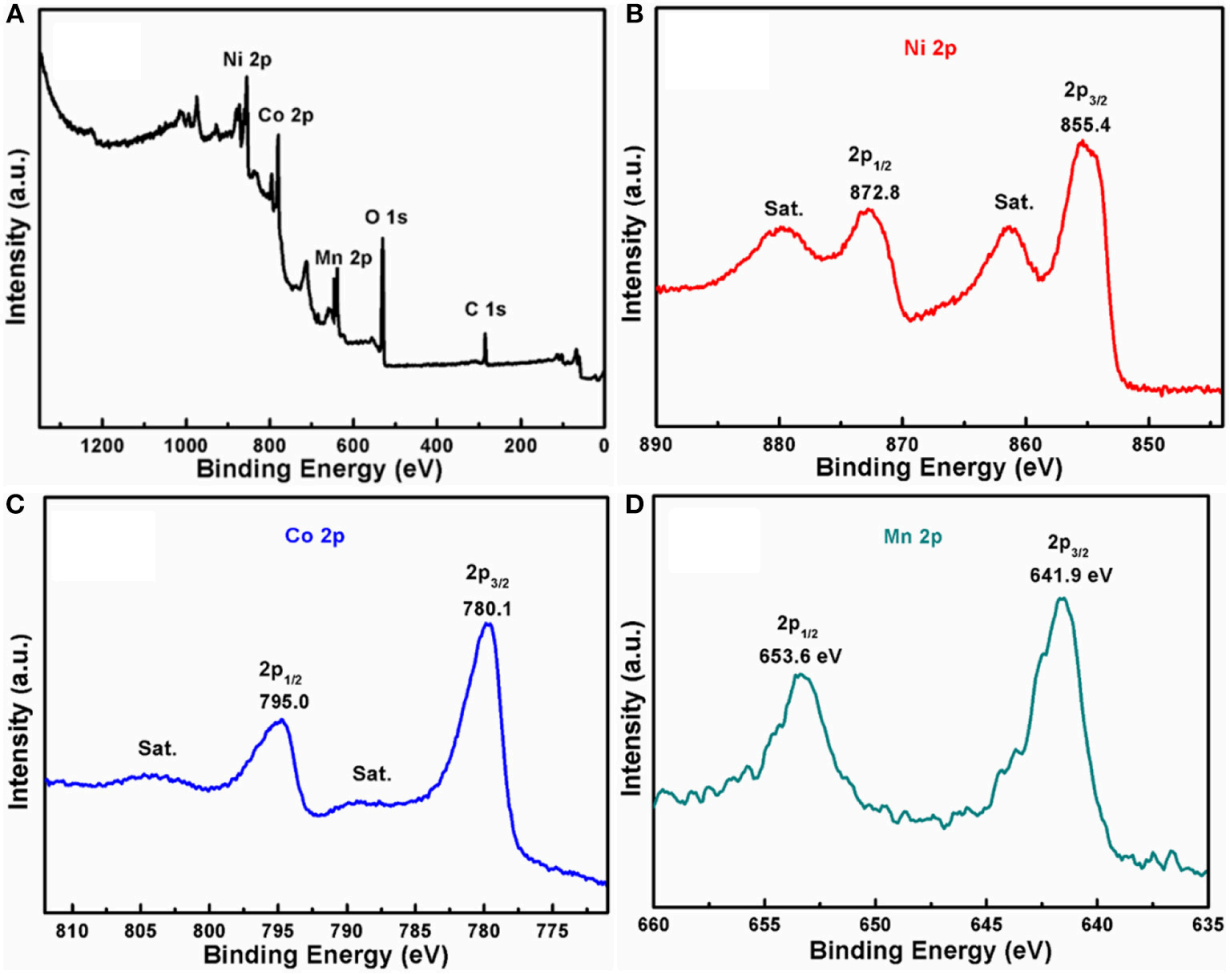
**(A)** XPS survey spectrum; **(B–D)** high-resolution Ni 2p, Co 2p, and Mn 2p XPS spectra of the as-made MnCo_2_O_4.5_@NiCo_2_O_4_.

XPS measurement was employed to reveal the composition and surface chemical states of the MnCo_2_O_4.5_@NiCo_2_O_4_, and the detailed results are shown in Figure 6. It can be clearly seen from survey spectrum (Figure 6A) that the MnCo_2_O_4.5_@NiCo_2_O_4_ consists of O, Ni, Co, and Mn elements, being consistent with EDS results (Figure 3D). The high resolution XPS spectrum of Ni 2p (Figure 6B) reveals that two obvious shakeup satellites (indicated as “*Sat*.”) are close to two spin orbit doublets at 855.4 and 872.8 eV, which represents the Ni 2p_3/2_ and Ni 2p_1/2_ splitting in Ni^2+^ chemical state, respectively (Hareesh et al., 2016; Sun et al., 2016; Wang et al., 2017; Zhang et al., 2018b). In the Co 2p XPS spectrum, the spin orbit splitting to the Co 2p_1/2_ (795.0 eV) and Co 2p_3/2_ (780.1 eV) reaches 14.9 eV (Figure 6C), indicating the co-existence of Co^2+^ and Co^3+^ in MnCo_2_O_4.5_@NiCo_2_O_4_ (Wu et al., 2015; Liu et al., 2017; Wang et al., 2017). Likewise, the peaks of Mn element locating at 641.9 eV and 653.6 eV in Figure 6D consistent with Mn 2p_3/2_ and Mn 2p_1/2_, respectively (Hui et al., 2016; Liu et al., 2017; Zhao et al., 2018; Zhu et al., 2018).

**Figure 7:**
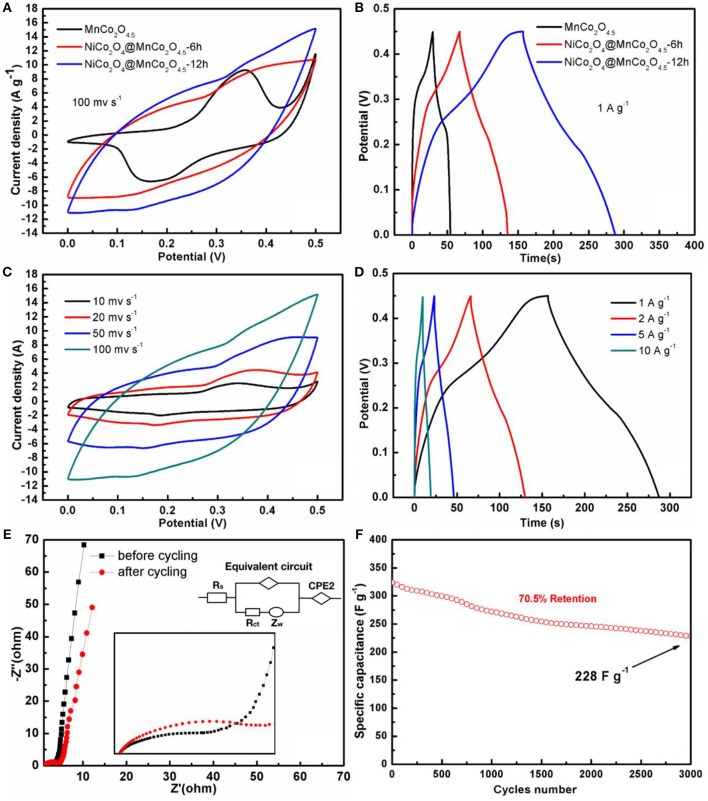
**(A,B)** CV and GCD curves of the samples; **(C)** CV curves of MnCo_2_O_4.5_@NiCo_2_O_4_*-*12h sample at different scan rates; **(D)** GCD curves of MnCo_2_O_4.5_@NiCo_2_O_4_*-*12h sample at different current densities. **(E)** Electrochemical impedance spectrum of MnCo_2_O_4.5_@NiCo_2_O_4_*-*12h sample before and after cycling. (Inset) Magnification of the electrochemical impedance spectrum at low frequency **(F)** Cycling performance of MnCo_2_O_4.5_@NiCo_2_O_4_*-*12h at the current density of 1 A g^−1^.

The electrochemical performance of the samples was analyzed by CV and GCD measurements. Figures 7A,B illustrates the CV and GCD curves of the three different electrodes at the same scan rate (100 mV S^−1^) and current density (1 A g^−1^), respectively. MnCo_2_O_4.5_@NiCo_2_O_4_-12h displays better rate performance with lower distortion compared with MnCo_2_O_4.5_@NiCo_2_O_4_-6h (Figure 7B). This is possibly due to the mass increasing of NiCo_2_O_4_ and the lower mass transport resistance within smaller interstitial regimes between the electrolyte and electrode materials. In Figure 7C, the CV curves of MnCo_2_O_4.5_@NiCo_2_O_4_-12h exhibit a quasi-reversible oxidative and reductive shape, indicating an ideal capacitance characteristic and a good reversibility of the architectures with good electronic conductivity and low internal resistance (Zhu et al., 2015; Hui et al., 2016; Sun et al., 2016; Wang et al., 2017). With the increment of scan rates, slight distortion of curves is observed, implying the favorable rate ability. The galvanostatic charge-discharge results of MnCo_2_O_4.5_@NiCo_2_O_4_-12h are presented in Figure 7D. Similarly, pseudo-capacitive behaviors are observed and the shape of GCD curves is not triangular ones which are believed to be pure electrical double layer capacitors (EDLCs).

The symmetrical shapes of the charge side and discharge side indicate the good reversibility of the synthesized composites. The MnCo_2_O_4.5_@NiCo_2_O_4_-12h composites (Figure 7D) have a higher specific capacitance of 325 F g^−1^ (146 C g^−1^) than that of MnCo_2_O_4.5_@NiCo_2_O_4_-6h [162.5 F g^−1^ (73 C g^−1^), Figure 7B] at the same discharge current density (1 A g^−1^), which is owning to its higher mass ratio of active NiCo_2_O_4_. Compared with pristine MnCo_2_O_4.5_, the electrochemical performance of synthesized core-shell composites is greatly improved based on the following two effects: MnCo_2_O_4.5_@NiCo_2_O_4_-12h has larger accessible surface area from loose NiCo_2_O_4_ shell layer which is built with isolated free-standing nanowires with smaller diameters; and shorter ion diffusion distance from smaller free-standing NiCo_2_O_4_ nanowires and interstitial regime between nanowires as well.

Figure 7E depicts the electrical impedance spectroscopy (EIS) of the composites before and after 3,000 cycling tests. The inset is magnified spectrum at low impedance region. The impedance spectrum is a semicircle parts at the high frequency and a linear curve at the low frequency. The intercept of the curves on the real axis reveals the total value of the ohmic resistance of electrolyte added with the resistance of the active materials. After cycling test, the radius of the semicircle increased obviously while first intercept and slope of the linear part change slightly. The apparent increasing of semicircle radius means more difficult for charge transfer of active species inside the electrode materials. The minor increasing of first intercept and decreasing of slope indicate higher contacting impedance and mass transport resistance after cycling tests. All the changes are derived from the potential structural damage of electrode materials during cycling tests. However, the overall capacitance retention is still up to 70.5% after 3,000 cycling tests (Figure 7F), which means this damage over cycling is at low level. This good cycling ability of MnCo_2_O_4.5_@NiCo_2_O_4_ is attributed to its unique one-dimensional structure. The one-dimensional structure has much free spaces between the nanowires, being able to accommodate the volume change of the materials during cycling and maintain the activity of the materials at high level.

## Conclusions

The MnCo_2_O_4.5_@NiCo_2_O_4_ composites with core-shell structure has been successfully synthesized with a facile and green hydrothermal method for high performance supercapacitors. The nanostructures, morphology and electrochemical performance of the composites are fully demonstrated. The MnCo_2_O_4.5_@NiCo_2_O_4_ composites exhibit remarkable capacitive performance with maximum capacitances of 325 F g^−1^ (146 C g^−1^) and retention 70.5% of the initial capacitance after 3,000 cycles at 1 A g^−1^. This excellent electrochemical performance of MnCo_2_O_4.5_@NiCo_2_O_4_ composites are attributed to the unique core-shell double spinel structures with shell built of isolated free-standing nanowires. The free spaces between nanowires are able to provide channels for mass transfer of active species and accommodate the volume change during cycling.

## Author Contributions

YXZ and YSY designed the research. WCH, XLL, and XYL performed the experiments. NL and TL carried out the partial experimental characterization. All authors incorporated in the discussion of experimental results.

## Conflict of Interest Statement

The authors declare that the research was conducted in the absence of any commercial or financial relationships that could be construed as a potential conflict of interest.
