# Supplementary material for: Facile Synthesis of Manganese Cobalt Oxide/Nickel Cobalt Oxide Composites for High-Performance Supercapacitors
Source: Front Chem. 2019 Jan 17;6:661. doi: 10.3389/fchem.2018.00661 (PMC6344439; doi:10.3389/fchem.2018.00661)
Supplement: Supplementary file 1 [file Table_1.DOCX]

**Supplementary Information**

**FIGURE S1 |** (A-B) SEM images of MnCo_2_O_4.5_ nanowires at different length scales.


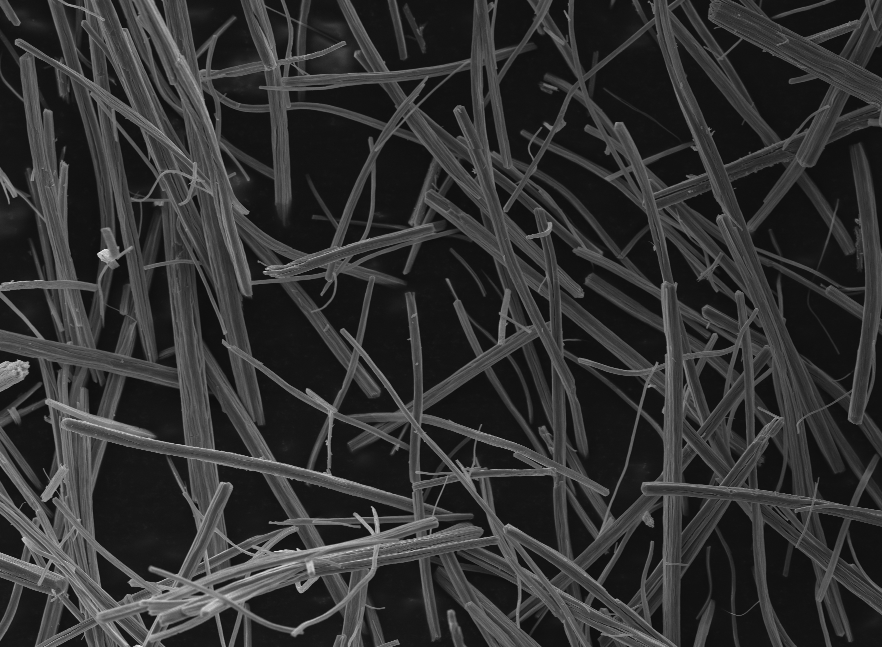


**4 μm**

**(A)**


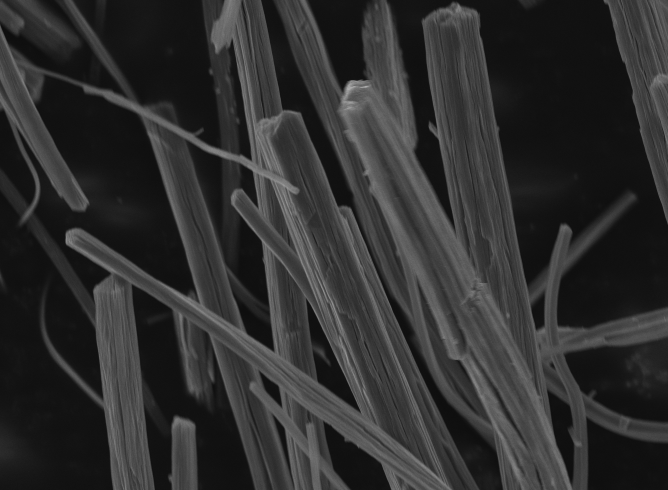


**1 μm**

**(B)**
